# Supplementary material for: Comparative multiomics analysis of cell physiological state after culture in a basket bioreactor
Source: Sci Rep. 2022 Nov 23;12:20161. doi: 10.1038/s41598-022-24687-4 (PMC9686226; doi:10.1038/s41598-022-24687-4)
Supplement: Supplementary file 1 — Supplementary Information 1. [file 41598_2022_24687_MOESM1_ESM.zip › raw data/Transcriptomic raw data/7.AS/2.dasplot/TE1vsTC1/SE/9_TRAPPC13_NW_023666049.1_10487132_10487176_+@NW_023666049.1_10490258_10490275_+@NW_023666049.1_10492952_10493085_+.pdf]

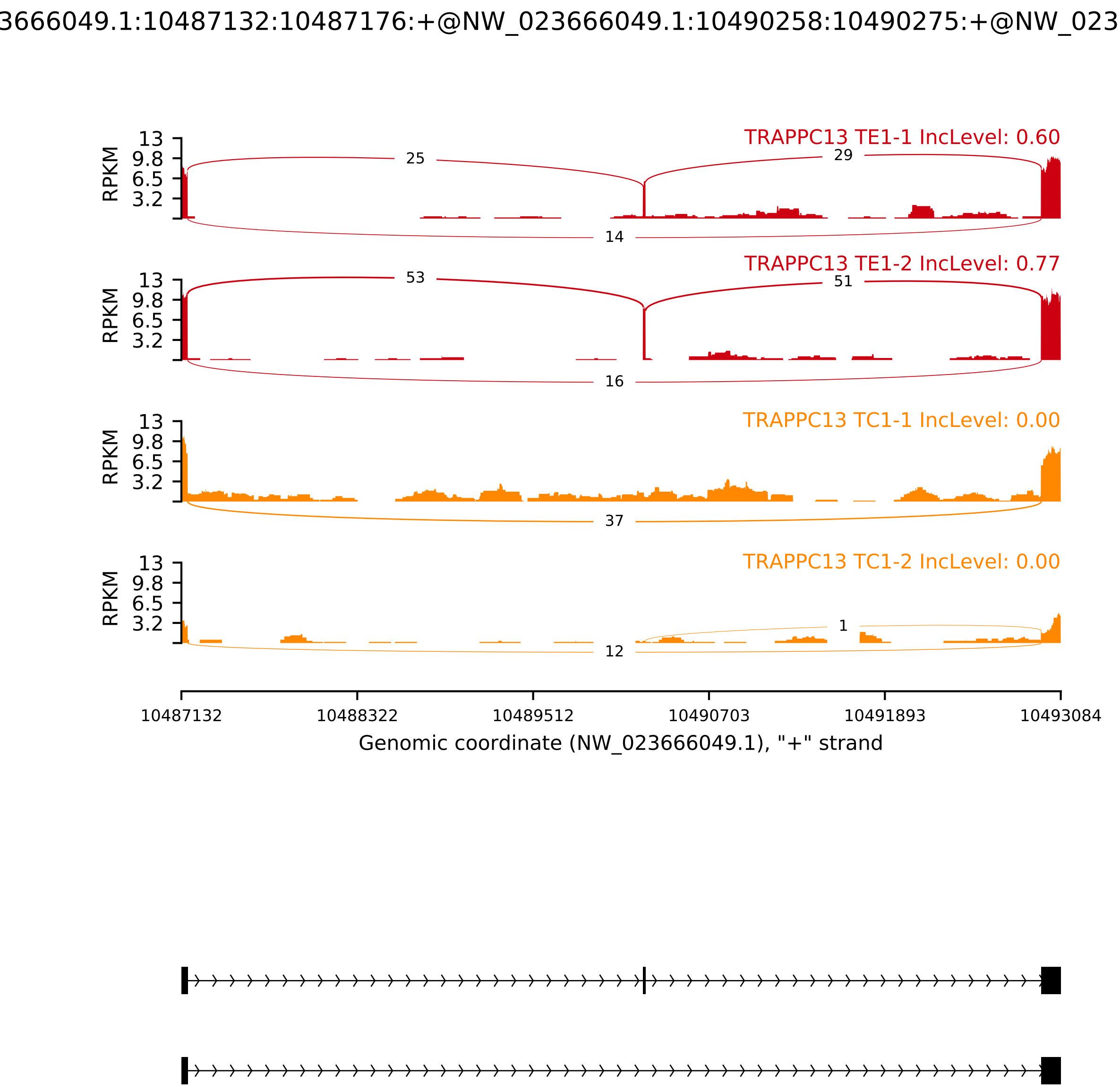

3666049.1:10487132:10487176:+@NW_023666049.1:10490258:10490275:+@NW_023666049.1:10492952:1049
TRAPPC13 TE1-1 IncLevel: 0.60
TRAPPC13 TE1-2 IncLevel: 0.77
TRAPPC13 TC1-1 IncLevel: 0.00
TRAPPC13 TC1-2 IncLevel: 0.00
RPKM
13
9.8
6.5
3.2
25
29
14
53
51
16
37
1
12
10487132
10488322
10489512
10490703
10491893
10493084
Genomic coordinate (NW_023666049.1), "+" strand
